# Kv beta complex facilitates exercise-induced augmentation of myocardial perfusion and cardiac growth

**DOI:** 10.3389/fcvm.2024.1411354

**Published:** 2024-06-24

**Authors:** Sean M. Raph, Ernesto Pena Calderin, Yibing Nong, Kenneth Brittian, Lauren Garrett, Deqing Zhang, Matthew A. Nystoriak

**Affiliations:** Center for Cardiometabolic Science, Department of Medicine, Division of Environmental Medicine, University of Louisville, Louisville, KY, United States

**Keywords:** cardiovascular, physical activity, vascular smooth muscle, ion channels, hypertrophy

## Abstract

The oxygen sensitivity of voltage-gated potassium (Kv) channels regulates cardiovascular physiology. Members of the Kv1 family interact with intracellular Kvβ proteins, which exhibit aldo-keto reductase (AKR) activity and confer redox sensitivity to Kv channel gating. The Kvβ proteins contribute to vasoregulation by controlling outward K^+^ currents in smooth muscle upon changes in tissue oxygen consumption and demand. Considering exercise as a primary physiological stimulus of heightened oxygen demand, the current study tested the role of Kvβ proteins in exercise performance, exercise-induced adaptations in myocardial perfusion, and physiological cardiac growth. Our findings reveal that genetic ablation of Kvβ2 proteins diminishes baseline exercise capacity in mice and attenuates the enhancement in exercise performance observed after long-term training. Moreover, we demonstrate that Kvβ2 proteins are critical for exercise-mediated enhancement in myocardial perfusion during cardiac stress as well as adaptive changes in cardiac structure. Our results underscore the importance of Kvβ proteins in metabolic vasoregulation, highlighting their role in modulating both exercise capacity and cardiovascular benefits associated with training. Furthermore, our study sheds light on a novel molecular target for enhancing exercise performance and improving the health benefits associated with exercise training in patients with limited capacity for physical activity.

## Introduction

Physical activity is the primary physiological stimulus of increased myocardial oxygen demand and consumption. During normal exercise, myocardial O_2_ consumption can increase by up to 6-fold (1–3). To meet the metabolic requirements of the exercise-conditioned heart, the vasculature undergoes functional adaptations to facilitate greater oxygen delivery during stress (4). Accordingly, human studies show increased myocardial perfusion and greater coronary flow reserve following exercise training (5–7). The results of animal studies also indicate that physical conditioning increases maximal myocardial perfusion (8–11). Nevertheless, how repetitive exercise over time promotes an increase in myocardial perfusion and its relationship with physical performance is unclear.

Physiological increases in O_2_ demand, such as those that occur during exercise, require instantaneous increases in myocardial perfusion via coronary arterial and arteriolar vasodilation. Our group and others have shown that this effect requires acute activation of voltage-gated potassium (Kv1) channels in coronary arterial smooth muscle cells, which thereby evokes hyperpolarization, reduction of intracellular [Ca^2+^]_I_, and relaxation (12–17). Native Kv1 channels of the vasculature are comprised of an α tetrameric pore domain that associates with a heterotetrameric Kvβ intracellular domain (18, 19). The Kvβ proteins are functional aldo-keto reductases that differentially regulate Kv gating upon binding oxidized and reduced cytosolic pyridine nucleotides [i.e., NAD[P(H)] (20, 21). This redox-sensing mechanism likely contributes to the maintenance of oxygen delivery and cardiac function during stress (22). Moreover, whereas the native Kvβ complex consists of functionally distinct Kvβ1 and Kvβ2 proteins, genetic models that modify Kvβ stoichiometry (increasing the Kvβ1:β2 ratio) effectively disrupt the relationship between myocardial oxygen demand and perfusion, and lead to systolic impairment during catecholamine-induced stress (17).

Recent work reinforces the view that myocardial perfusion is a vital limiting factor of cardiorespiratory fitness in advanced age (23). Therefore, the identification of novel targets that participate in the coupling of blood supply with oxygen demand in the heart and peripheral tissues holds promise for improving strategies that enhance the ability to exercise as well as the myriad cardiovascular health benefits associated with recurrent physical activity. Based on the abovementioned findings, we hereby tested the hypotheses that Kvβ proteins are critical for exercise performance, facilitate enhanced myocardial perfusion of the exercise-conditioned heart, and support the physiologic cardiac growth response elicited by structured physical activity.

## Methods

### Ethical approval

All animal protocols were conducted in accordance with the Guide for the Care and Use of Laboratory Animals as approved by the Institutional Animal Care and Use Committee at the University of Louisville (protocol no. 21969).

### Animals

Wild-type (wt; 129SvEv) and homozygous Kvβ2^−/−^ (129SvEv background) mice, aged 12–20 wks, were used for this study. For a select set of experiments, double transgenic sm22α-rtTA:TRE-Kcnab1 (rtTA–Kcnab1) and littermate single transgenic sm22α-rtTA (rtTA) mice (FVB background) were also used. The sm22α-rtTA and TRE-Kcnab1 transgenes allow overexpression of Kvβ1 in smooth muscle upon treatment of mice with doxycycline (2 mg/ml in drinking water; started 10 days prior to, and maintained throughout, exercise protocols) (24). To avoid confounding results due to the disparate effects of sex hormones on vascular K^+^ channel expression and exercise performance, only male mice were used in this study, with the exception of MCE analyses of myocardial perfusion. All mice were bred in-house and maintained in a temperature-controlled room on a 12:12 light: dark cycle with *ad libitum* access to food and water. At termination, mice were euthanized by intraperitoneal injection of sodium pentobarbital (150 mg/kg) followed by thoracotomy and heart removal. Tissues were immediately excised for post-mortem tissue analyses.

### Exercise capacity tests and training protocols

All mice underwent a 2-day treadmill familiarization period (one familiarization session per day) before evaluating baseline exercise capacity on the third, following day. For familiarization sessions, mice were placed on a lane of an Exer 3/6 motorized treadmill (Columbus Instruments, Columbus, OH) that was set at a speed of 0 m/min for 10 min, at a 0° incline grade relative to horizontal, and the shock grid was set to an intensity of 0.3–0.7 mA (2 Hz). After 10 min, the treadmill speed was slowly increased from 0 to 10 m/min for 12 min.

**Figure 1:**
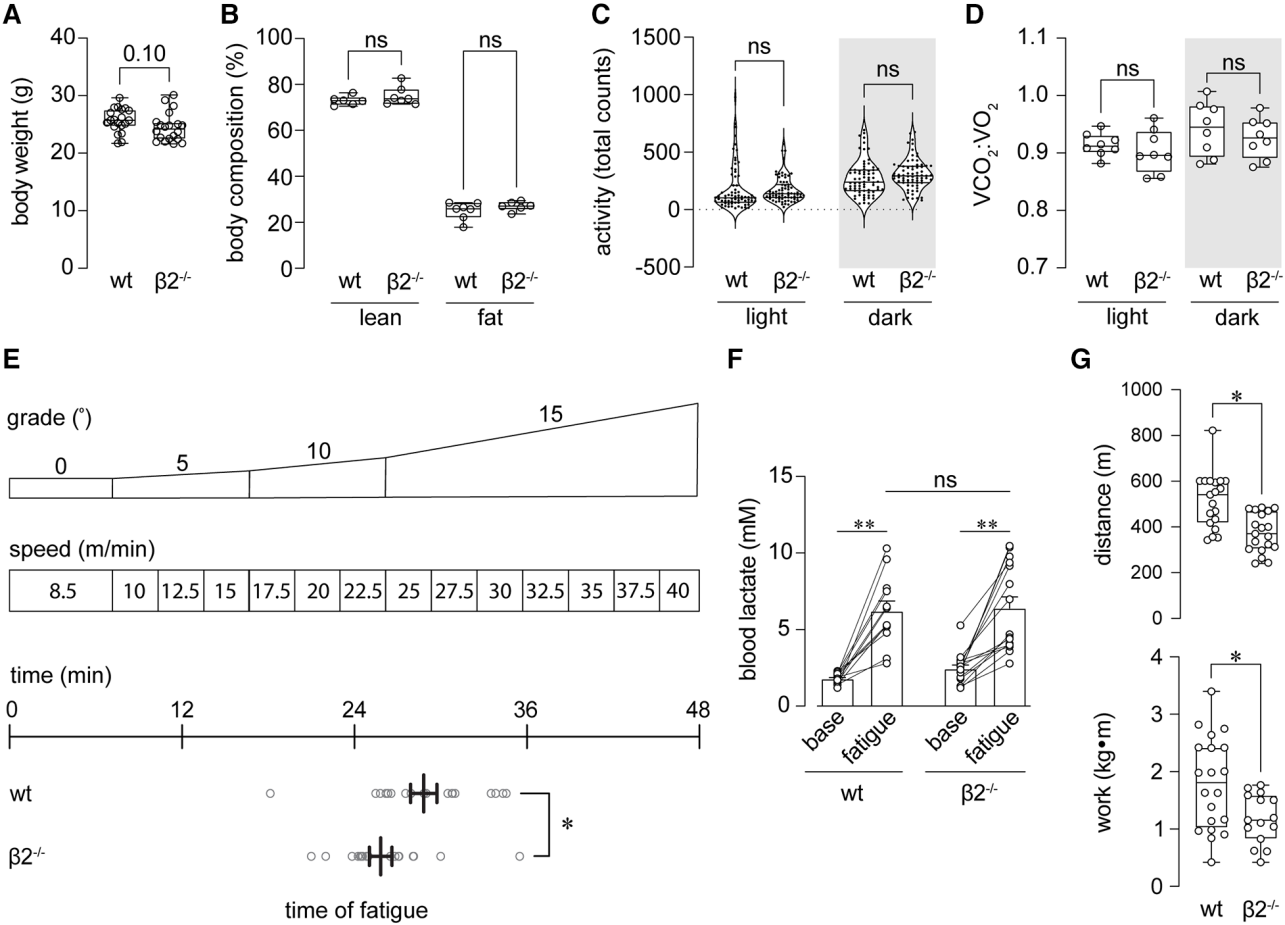
Mice lacking Kvβ2 exhibit reduced baseline exercise capacity. (**A,B**) Summary plots showing body weight at baseline (pre-training; A) and lean and fat mass body compositions (**B**) measured in wild-type (wt) and β2^−/−^ mice. (**A**) *p* = 0.10, unpaired *t*-test, *n* = 20 mice each; (**B**) ns: *p* > 0.05, Kruskal-Wallis test with Dunn's multiple comparisons test. (**C**) Total activity (sum of beam break counts over 10 min intervals for 24 h) during light and dark phases, as indicated, measured in wt and β2^−/−^ mice; ns: *p* > 0.05, one-way ANOVA with Šídák's multiple comparisons test. (**D**) Summary plot showing mean respiratory exchange ratios (VCO_2_:VO_2_) measured in wt and β2^−/−^ mice during light and dark phases. ns: *p* > 0.05, Brown-Forsythe ANOVA test with Dunnett's multiple comparisons test; *n* = 8 mice each. (**E**) Schematic (*top*) showing exercise capacity test protocol in which mice are subjected to increasing grade and speed on a treadmill to exhaustion (see Methods); and, horizontal plot showing time of exhaustion for wt and β2^−/−^ mice. *p < 0.05, unpaired *t*-test, *n* = 19–20 mice. (**F**) Blood lactate concentration measured before capacity test (baseline) and immediately upon fatigue in wt and β2^−/−^ mice. **p < 0.01, ns: *p* > 0.05, two-way ANOVA with Šídák's multiple comparisons test, *n* = 12–16 mice. (**G**) Summary of distance (top) and work performed (bottom) at exhaustion in wt and β2^−/−^ mice. *p < 0.05; *n* = 15–20 mice.

Prior to exercise capacity tests (ECT), body weights were recorded and blood lactate levels were measured at rest with a hand-held Lactate Plus sensor (Nova Biomedical, Waltham, MA, USA) via a distal tail clip. Mice were then placed on the treadmill and allowed to rest for 3–5 min before starting the ECT. The treadmill shock grid remained active (settings as described above) throughout the ECT. Each ECT consisted of an initial warmup period (9 min, 8.5 m/min, 0° grade), followed by 2.5 m/min increment speed increases every 3 min and 5° grade increases at the 9th, 18th, and 27th minute (see Figure 1E). Using this protocol, the amount of work performed (vertical distance ×  body mass) increases incrementally throughout the procedure. Note that this design of high intensity, short duration ECT (Bruce protocol) is well established for assessment of exercise performance as a function of VO_2_ max and anaerobic crossover points (assessed by initial and final blood lactate concentration from venous tail blood and respiratory quotient through indirect calorimetry) (25, 26). The exhaustion criteria used to determine the fatigue point during the ECT and during regular exercise sessions (see below) has been previously described (27) and were as follows: (1) the mouse is subjected to >10 s of consecutive shocks on the grid, and/or (2) mouse spends >50% of time spent receiving shocks, for any rolling time interval, and/or (3) upon manual prodding, the mouse displays a lack of motivation to resume running. After recording the fatigue point for each animal (distance, time, and maximal speed achieved), the individual shock grid lane was turned off and the mouse was removed from the treadmill for immediate measurement of post-fatigue blood lactate levels. Mice were then returned to their home cage with *ad libitum* access to food and water. Age-matched wt, β2^−/−^, rtTA, and rtTA–Kcnab1 mice were randomly assigned into sedentary and exercise groups. All mice undergoing exercise training had a 72 h rest period before starting the 4 wk training regimen.

**Figure 2:**
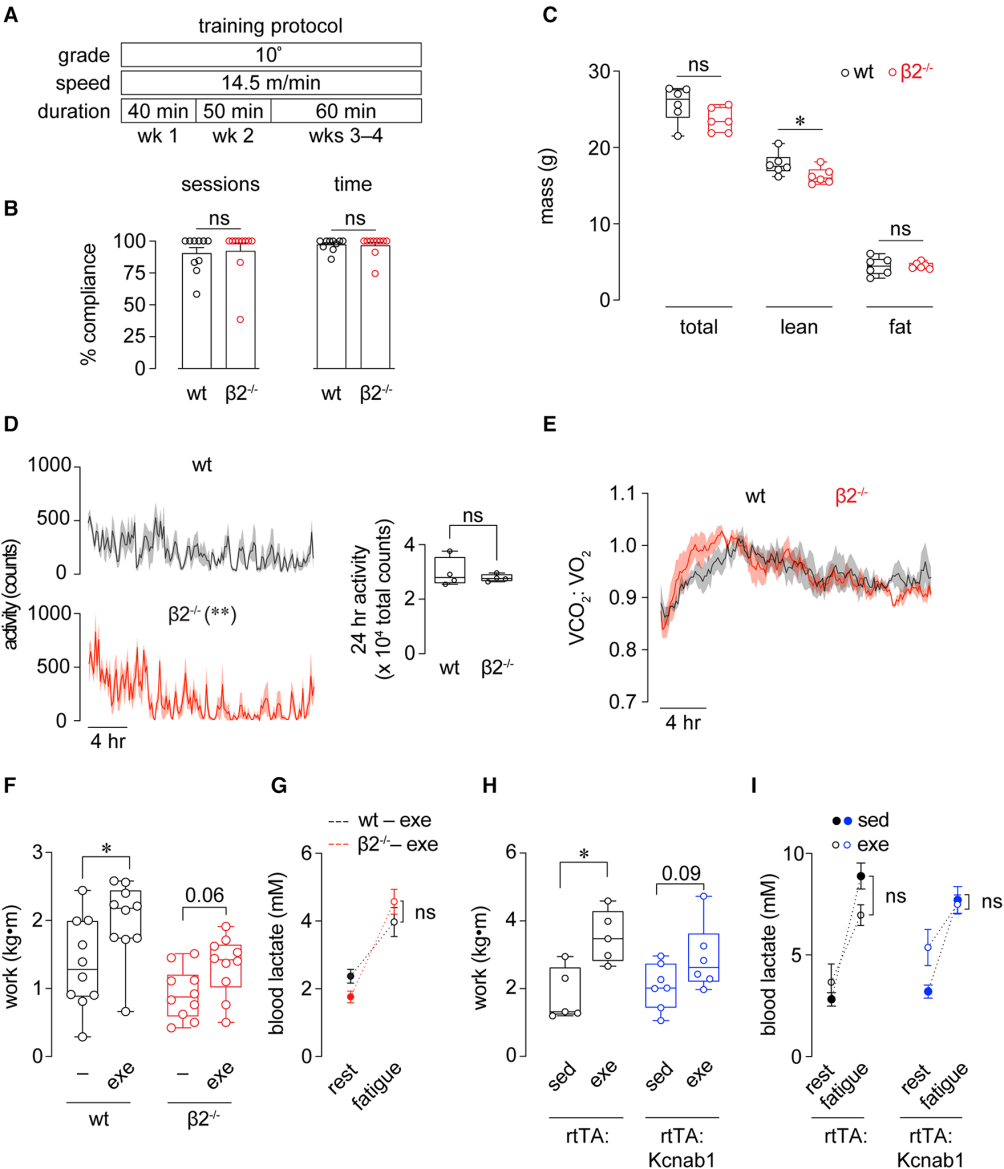
Improvements in exercise capacity following training are facilitated by Kvβ proteins. (**A**) Time course of 4 wk exercise training protocol used for wt and β2^−/−^ mice. (**B**) Compliance of wt and β2^−/−^ mice to the 4 wk exercise training protocol, recorded as either the percent of total sessions completed, or percent time (min) completed. ns: *p* > 0.05, Mann Whitney test; *n* = 10 mice each. (**C**) Total, lean, and fat body mass measured in wt and β2^−/−^ mice following completion of the 4 wk exercise training protocol. *p < 0.05, ns: *p* > 0.05, Mann Whitney test, *n* = 6 mice each. (**D**) Activity plots showing beam break counts recorded in 10 min intervals over a 24 h period in wt and β2^−/−^ mice following completion of exercise training protocol. **p < 0.01, two-way ANOVA. Inset shows 24 h summed activities measurements. ns: *p* > 0.05, unpaired *t*-test. (**E**) Mean (line) ± SE (shading) 24 h respiratory exchange ratio (VCO_2_:VO_2_) measured in wt and β2^−/−^ mice following completion of training protocol. *p* > 0.05, two-way RM ANOVA; *n* = 4 mice each. (**F**) Work performed to exhaustion in exercise capacity tests before (-) and after (exe) exercise training period in wt and β2^−/−^ mice. wt: *p < 0.05, β2^−/−^: *p* = 0.06, paired *t*-test, *n* = 10 mice each. (**G**) Blood lactate concentrations measured at rest (before capacity test) and after fatigue in exercise trained wt and β2^−/−^ mice. ns: *p* > 0.05, two-way RM ANOVA, *n* = 10 mice each. (**H**) Work performed to exhaustion in exercise capacity tests in sedentary (sed) and exercised (exe) sm22α-rtTA and sm22α-rtTA:TRE-Kcnab1 transgenic mice. *p < 0.05, paired *t*-test, *n* = 10 mice each. (**I**) Blood lactate concentrations measured at rest (before capacity test) and after fatigue in sedentary and exercise-trained rtTA and rtTA:Kcnab1 mice. ns: *p* > 0.05, two-way RM ANOVA, *n* = 5–7 mice.

_­_Regular forced treadmill running sessions were administered 5 d/wk (Mon–Fri) for 4 wks. Each session consisted of a 12 min warmup period (5 m/min, 10° grade), immediately followed by either 40 min (wk 1), 50 min (wk 2), or 60 min (wks 3–4) of running. For the running period, the treadmill speed was assigned at 50% (wt, β2^−/−^) or 70% (rtTA, rtTA-Kcnab1) of the average maximal speed attained during the initial ECT before fatigue (see Figure 2). Any animals meeting criteria for fatigue, as described above, were removed from the treadmill for that day and returned to the training program on the next day. A cooldown period (5 m/min, 10° grade) was allowed before returning mice to their home cage with *ad libitum* access to food and water. Following 4 wks of exercise training, mice underwent a second ECT protocol to assess changes in exercise capacity.

### Body mass composition analysis

Adipose content and lean mass were determined using a small animal Lunar PIXIMus x-ray densitometer (Dexascan). Mice were anesthetized with 2% isoflurane and subjected to Dexascan measurements of lean and fat mass, expressed as absolute mass and percent of total body weights, as previously described (28).

### Metabolic phenotyping

Mice were singly-housed in cages (25 × 15  ×  24 cm dimensions; 9 L) with *ad libitum* access to food and water, and metabolic and behavioral information was continuously recorded using a TSE PhenoMaster/LabMaster high-throughput phenotyping platform (TSE Systems, Bad Homburg, Germany). Indirect gas calorimetry was performed using an airflow rate of 0.35 L/min and a sampling rate of 10 min with reference-air cage readings performed during each interval to account for changes in room air conditions. Recordings from each mouse were performed over a 24 h period to monitor oxygen consumption (VO_2_), carbon dioxide production (VCO_2_) (both normalized to body weight), respiratory exchange ratio (VCO_2_:VO_2_), food and water consumption, and movement.

### *In vivo* measurements of myocardial perfusion and cardiac function

In vivo measurements of myocardial perfusion and cardiac structure and function were performed as previously described (22). Briefly, mice were anesthetized with isoflurane (3% induction, 1%–2% maintenance; supplemental O_2_ delivered at 1 L/min) and placed on a heating platform in the supine position. A small incision was made on the right side of the neck for placement of a jugular venous catheter (sterile PE-50 tubing, prefilled with heparinized saline; 50 units/ml) to deliver the contrast agent and drugs. For myocardial contrast echocardiography (MCE), lipid-shelled microbubbles were prepared by sonication of a decafluorobutane gas-saturated aqueous suspension of distearoylphosphatidylcholine (2 mg/ml) and polyoxyethylene-40-stearate (1 mg/ml). The microbubble suspension was diluted 1:10 in saline and infused intravenously at 20 μl/min. Imaging was performed using the nonlinear contrast module of a Vevo 3100 ultrasound system with a MX250 probe (center frequency 21 MHz) (FujiFilm VisualSonics, Toronto, ON, Canada). Using the burst replenishment function, ECG-triggered image acquisition was performed during (destruction) and following (replenishment) a 1 s high energy ultrasound pulse to destruct intravascular contrast agent within the acoustic field. At baseline, mice were treated with atropine (0.04 mg/kg; i.p.) and ≥10 sequences were recorded before and during administration of the β-adrenoceptor agonist dobutamine (10 μg/kg/min). Parallel measurements of cardiac structure and function were performed under each condition, as previously described using a MX550D probe (center frequency 40 MHz) (29). All data analyses and calculations of myocardial perfusion were conducted offline using VevoLAB (FujiFilm VisualSonics, Toronto, ON, Canada) and custom-written software (MATLAB; see below).

For MCE measurements, the acoustic gain was adjusted to obtain images with minimal myocardial contrast intensity signal in the absence of contrast agent infusion and maintained constant throughout the study. Long-axis images were acquired at a penetration depth of 15–20 mm (focus depth 7.5 mm). Images were analyzed to determine perfusion by fitting intensity data from the anterolateral myocardial region with an exponential function: y* = *A(1­–e^−βt^) where *y* is the signal intensity at time *t*, *A* is the signal intensity at plateau during the replenishment phase (reflects microvascular volume), and β is the slope of the initial replenishment phase and reflects the volume exchange frequency. Myocardial perfusion was estimated by compiling 3–5 image sequences per condition and fitting data to determine the product of β × relative blood volume (*A*: mean cavity signal intensity). All measurements were performed in a genotype/treatment-blinded fashion. A MATLAB program was used to compile burst data in VevoLAB-derived.csv outputs. After subtracting average background intensities, data were fit with an exponential function (described above). Data points >1.5 standard deviations from the best fit were excluded and a new fit was generated to determine *A*, β*,* and average cavity signal intensities from compiled datasets.

### Histology

At euthanasia, hearts were excised and flushed with 1M KCl in phosphate-buffered saline via cannulation of the aorta. Transverse mid-ventricular sections with the right and left ventricles visible were fixed with 10% neutral buffered formalin for 24 h. Following fixation, heart sections were transferred to 70% ethanol, embedded in paraffin, and sections (4 µm) were cut using a microtome. Following standard xylene/ethanol deparaffinization, antigen retrieval was performed with 0.1 M sodium citrate (pH 6.8) (Sigma), and slides were treated with Sudan Black to minimize autofluorescence. Sections were then stained with Alexa Fluor 555-conjugated wheat germ agglutinin (WGA) (Life Technologies) and Fluorescein-conjugated anti-isolectin B4 (Vector Biolabs) and mounted using SlowFade Gold Antifade mountant with DAPI (Life Technologies).

All image acquisition and analyses were performed in a blinded manner. Images were acquired from the left ventricular free wall (anterior and posterior) for Alexa Fluor 555 (WGA) and Fluorescein (Isolectin B4) using a Nikon Eclipse-Ti epifluorescence microscopy system with a 20 × objective lens. Myocytes in a cross-sectional view within the subendocardial region were chosen for analysis using the following criteria: (1) oblong-to-circular cell outline, with centric and round nuclei with capillaries (Isolectin B4 positive staining) surrounding the cardiomyocyte in cross-sectional (rather than oblique) view. For analysis of cross-sectional area, ≥100 cardiomyocytes were counted per animal.

### *In situ* proximity ligation

Arterial smooth muscle cells were obtained from dissected coronary arteries, including the left main and anterior descending arteries, as previously described (22). Briefly, arteries were incubated in digestion buffer containing (in mM): 140 NaCl, 5 KCl, 2 MgCl_2_, 10 HEPES, and 10 glucose, at pH 7.4, at 37 °C for 1 min. The buffer was subsequently exchanged for a solution containing papain (1 mg/ml; Worthington) and dithiothreitol (1 mg/ml; Sigma Aldrich) and incubated at 37 °C for 5 min with gentle agitation. The papain/dithiothreitol buffer was then replaced with digestion buffer containing collagenase H (1.25 mg/ml; Sigma Aldrich) and trypsin inhibitor (1 mg/ml; Sigma Aldrich) and incubated at 37 °C for 5 min with gentle agitation. The digested tissue underwent three washes with ice-cold enzyme-free digestion buffer and was then triturated with a flame-polished Pasteur pipette to liberate individual arterial smooth muscle cells.

Isolated coronary arterial smooth muscle cells were transferred onto glass microscope slides while in suspension and allowed to adhere (−20 min, room temperature). After adherence, the cells underwent a wash with phosphate-buffered saline (PBS) and were subsequently fixed in 4% paraformaldehyde for 10 min. To detect protein-protein interactions, an *in situ* proximity ligation assay (PLA) kit (Duolink; Sigma Aldrich) (30) was utilized following the manufacturer's instructions. Initially, the cells were blocked using Duolink blocking solution and then exposed to primary antibodies targeting Kv1.5 (Neuromab, 75–011; 1:50), Kvβ1 (Abcam, AB174508; 1:100), and Kvβ2 (Aviva Systems Biology, ARP37678_T100, 1:100) (29). The labeled Kv proteins were detected using oligonucleotide-conjugated PLA probe secondary antibodies (anti-rabbit PLUS, anti-mouse MINUS), followed by a reaction with PLA probe-specific oligonucleotides and ligase to produce circular nucleotide products at sites of probe-probe proximity (<40 nm). Subsequently, the cells were incubated for 100 min at 37 °C in a solution containing polymerase and fluorophore-tagged oligonucleotides for rolling circle amplification, concatemeric product generation, and fluorescent labeling. After washing, the slides were mounted with Duolink mounting media containing DAPI nuclear stain, and coverslips were sealed with nail polish. Fluorescent images were captured using a Keyence BZ-X800 All-in-One fluorescence imaging system. Images were analyzed using FIJI software (NIH, Bethesda, MD) to obtain counts of total fluorescent PLA puncta in each cell. Complete z-series images (0.5 μm step) for each cell were flattened using the z-project function, and the number of PLA-associated punctate particles for each cell was counted and normalized to the cell footprint area obtained from transmitted light images.

### Assessment of growth markers

Total RNA was isolated from hearts using an RNeasy Fibrous Tissue Isolation kit (Qiagen), and RNA purity was assured by treatments with proteinase K (Qiagen) and DNase (Qiagen). The quantity and quality of isolated RNA were determined using a NanoDrop 2000C spectrophotometer (Thermo Scientific). RNA was reverse-transcribed for the first strand cDNA synthesis with an RNA-directed DNA polymerase, the Avian Myeloblastosis Vi­rus (AMV) Reverse Transcriptase (Promega), and Oligo dT primers (IDT). Assays were performed in triplicate for each gene and condition, and real-time PCR amplification was performed using iTag Universal SYBR Green Supermix reagents (Bio Rad) on a 7900TH Fast Real-Time PCR System (Applied Biosystems). The threshold cycle (Ct) was obtained, and the relative transcript level was calculated using the 2-ΔΔCt method. The relative expression levels of target genes were normalized to that of the endogenous reference gene hypoxanthine phosphoribosyltransferase 1 (HPRT1) in each sample, and differences in mRNA expression in exercise groups were expressed as fold relative to respective sedentary controls. The sequences of primers used are: C/EBPβ3, Forward: 5’-ACGACTTCCTCTCCGACCTCT-3’, Reverse: 5’-AGGCTCACGTAACCGTAGTCG-3’; CITED4, Forward: 5’-CATGGACACCGAGCTCATC-3’, Reverse: 5’-CTGACCCCAGGTCTGAGAAG-3’; NFATC2, Forward: 5’-ACGGGAGTGACCCTCAAA-3’, Reverse: 5’-CTGACCCCAGGTCTGAGAAG-3’; HPRT1, Forward: 5’-AGGACCACTCGAAGTGTTGG-3’, Reverse: 5’-AGGGCATATCCAACAACAAAC-3’.

### Statistical analysis

Data were analyzed by Prism 10 software (GraphPad). Shapiro-Wilk tests were used to test normality. Normal data were compared using two-tailed *t*-tests for two groups (unpaired or paired depending on comparison), and one-way ANOVA with post-hoc tests for multiple comparisons, as indicated, of 3 or more groups. Two-way ANOVA and Mixed Effects were used to test for interactions in time, treatment, and/or genotype. Nonparametric tests were used for comparisons of non-normal datasets. Specific tests and associated *p* values for all comparisons are provided in Figure Legends; *p* < 0.05 was considered statistically significant. Data are presented as means ± standard error unless otherwise indicated. Box-and-whiskers plots show the median (line), 25th–75th percentiles (box), and range (whiskers).

## Results

### Loss of Kvβ2 proteins reduces baseline exercise capacity

We tested whether mice with global deletion of Kcnab2 (β2^−/−^) have differences in their baseline exercise capacity relative to wild-type (wt) mice. Before performing exercise capacity tests, we first evaluated whether loss of Kvβ2 proteins leads to overt changes in body weight, body mass composition, activity, or systemic metabolic activity in adult mice, as this could potentially impact the interpretation of exercise performance measures. As shown in Figure 1, the body weight of β2^−/−^ male mice trended lower (*p* = 0.10) relative to age- and sex-matched wt mice. However, no differences in lean or fat body mass were noted between naïve wt and β2^−/−^ mice (Figure 1B). Activity patterns during the inactive (light; 6 a.m.—6 p.m) and active (dark; 6 p.m.—6 a.m.) phases were also similar between β2^−/−^ and wt mice. Upon evaluation of baseline systemic metabolic activity, we observed no significant differences in oxygen consumption rate, carbon dioxide production rate, respiratory exchange ratio (a surrogate measure of whole-body substrate utilization), or energy expenditure measured during the inactive or active phases between β2^−/−^ vs. wt mice (Figure 1D and Table 1).

**Table 1:** Baseline systemic metabolic activity in wild-type and β2^−/−^ mice.

	Wild-type		β2^−/−^	
	Light	Dark	Light	Dark
VO_2_ (ml/h/kg)	3,211 ± 115	3,525 ± 93	3,365 ± 149	3,680 ± 175
VCO_2_ (ml/h/kg)	2,931 ± 121	3,310 ± 100	3,036 ± 143	3,403 ± 167
VCO_2_:VO_2_ (RER)	0.91 ± 0.01	0.90 ± 0.01	0.94 ± 0.02	0.93 ± 0.01
En. Exp. (Kcal/h/kg)	15.9 ± 0.6	17.6 ± 0.5	16.7 ± 0.8	18.3 ± 0.9

VO_2_, oxygen consumption rate; VCO_2_, carbon dioxide production rate; RER, respiratory exchange ratio; En. Exp.: energy expenditure. Values represent mean ± standard error over 12 h light or dark phase periods. *p* > 0.05. for each, β2^−/−^ light vs. wild-type light or β2^−/−^ dark vs. wild-type dark, one-way ANOVA with Šídák's multiple comparisons test, *n* = 8 each.

We subjected β2^−/−^ and age-matched wt mice to an established treadmill running protocol to assess exercise capacity as the level of work performed to exhaustion (27). Briefly, after a short warmup period, the mice ran at incrementally increasing treadmill speeds and incline grades (Figure 1E) to the point of meeting pre-defined fatigue criteria (see Methods). Using this protocol, the total time completed to fatigue was significantly lower among β2^−/−^ mice compared with wt mice (Figure 1E). To determine whether mice from both groups were meeting exhaustion criteria due to fatigue rather than behavioral differences that may arise due to β2 deletion, we measured blood lactate levels before capacity tests and immediately following exhaustion. As shown in Figure 1F, fatigued β2^−/−^ mice had lactate levels comparable to that measured in fatigued wt mice, suggesting that β2^−/−^ were reaching exhaustion at significantly earlier time points than wt mice. Accordingly, β2^−/−^ mice ran less distance and performed less work than wt mice (Figure 1G). These data indicate that the loss of Kvβ2 proteins attenuates baseline exercise capacity in mice.

### Loss of Kvβ2 proteins attenuates training-induced improvement in exercise performance

After evaluating baseline exercise capacity, we subjected mice to a chronic exercise training protocol in which mice ran at −50% of their initial maximal speed on a treadmill 5 d/wk for 4 wks (see Figure 2A). At this moderate intensity, β2^−/−^ mice completed the 4^−^wk exercise training protocol with compliance similar to that of wt mice, as supported by no differences observed in the number of sessions completed or total time completed between groups (Figure 2B). After the 4^−^wk training period, no differences in body weight or fat mass were noted between groups. A modest but significant reduction in lean mass was observed in exercise-trained β2^−/−^ mice compared with exercise-trained wt mice (Figure 2C). Whereas diurnal activity patterns were significantly altered in exercise-trained β2^−/−^ mice, the total 24-hr activity levels were not different from exercise-trained wt mice (Figure 2D). In addition, diurnal RER patterns in exercise-trained β2^−/−^ mice were similar to that measured in exercise-trained wt mice (Figure 2E).

After training, wt mice exhibited a significant improvement in the total work performed to exhaustion in follow-up exercise capacity tests (Figure 2F). Nonetheless, despite completing the same level of exercise training, exercise-trained β2^−/−^ mice failed to show significant improvement in capacity relative to baseline assessments (Figure 2F). Similar to observations in baseline capacity tests, no differences in blood lactate at fatigue were observed between β2^−/−^ and wt exercise-trained mice (Figure 2G), altogether suggesting that Kvβ2 proteins facilitate adaptations in exercise performance following long-term training.

To test whether this role of Kvβ proteins could be attributed to their known role in regulating organ perfusion (17), we repeated these experiments in transgenic mice with inducible over-expression of Kvβ1 proteins in smooth muscle cells (rtTA-Kcnab1 see Methods, Animals). Note that this model enables a smooth muscle cell-specific reduction of the Kvβ2:β1 ratio, as demonstrated previously (17, 24). Similar to observations in wt 129SvEv mice (genetic background of β2^−/−^ mice), single transgenic rtTA mice (FVB background) exhibited robust improvement in work performed in exercise capacity tests after training (Figure 2H). Whereas rtTA-Kcnab1 mice showed a trend towards enhanced work after training, this change did not reach statistical significance, suggesting that the vascular Kvβ2:β1 ratio contributes to the training-induced improvement in exercise capacity in mice.

### Enhanced myocardial perfusion during stress following exercise training requires Kvβ2 proteins

**Figure 3:**
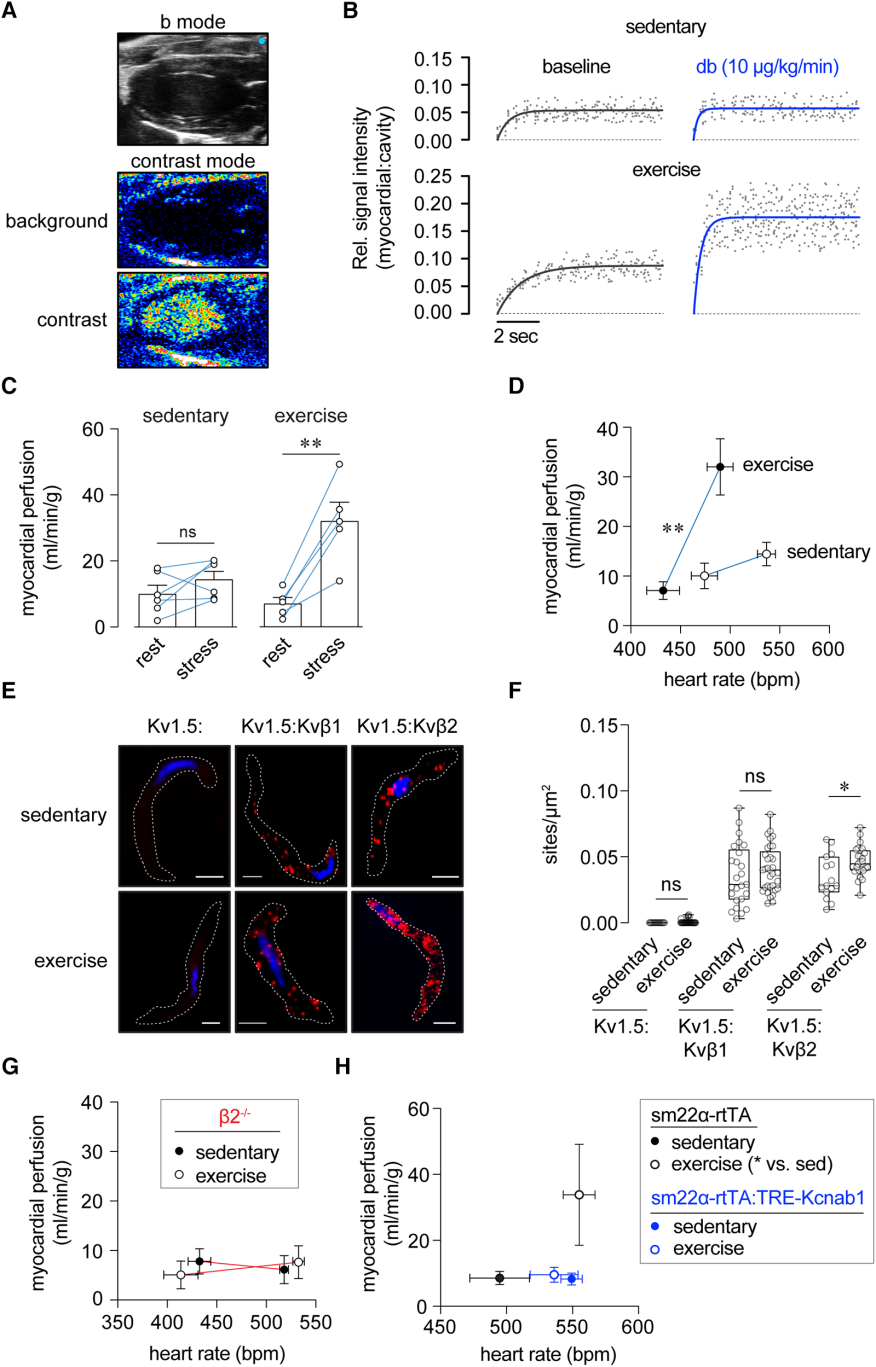
Enhanced myocardial perfusion during stress following exercise training requires Kvβ2 proteins. (**A**) Exemplary B-mode and nonlinear contrast images (before and during contrast agent infusion). (**B**) Representative replenishment plots showing time course and magnitude of contrast intensity following destruction phase in sedentary and exercise-trained mice at rest (baseline) and stress (dobutamine, 10 μg/kg/min, i.v.). Data are fit with a one-phase exponential growth function: Y = A(1-e^βx^). (**C**) Summary plot of myocardial perfusion at rest and during dobutamine-induced stress in sedentary and exercise-trained mice. **p < 0.01, ns: *p* > 0.05, paired *t*-test. *N* = 5–6 mice. (**D**) Relationships between myocardial perfusion and HR in anesthetized sedentary and exercise-trained mice at rest and stress. **p < 0.01, mixed effects analysis on perfusion: HR, *n* = 5–6 mice. (**E**) Representative proximity ligation images showing positive sites of interaction (red) between Kv1.5 and Kvβ1, and Kv1.5 and Kvβ2 in coronary arterial smooth muscle cells from sedentary and exercised mice. Singly labelled cells for Kv1.5 are shown as a control. DAPI nuclear stain is shown in blue. Scale bars represent 10 μm. (**F**) Summary plot showing the number of PLA-positive sites/μm^2^ of cell footprint area in coronary arterial smooth muscle cells from sedentary and exercise-trained mice labelled for Kv1.5 alone, Kv1.5 and Kvβ1, and Kv1.5 and Kvβ2. *p < 0.05, ns: *p* > 0.05, Mann Whitney test. (**G**) Relationships between myocardial perfusion and HR in sedentary and exercise-trained β2^−/−^ mice at rest and stress. *p* > 0.05, mixed effects analysis on perfusion:HR, *n* = 5–6 mice. (**H**) Relationships between myocardial perfusion and HR in sedentary and exercise-trained rtTA and rtTA:Kcnab1 mice at rest. *p < 0.05, one-way ANOVA with Dunnett's multiple comparisons test on perfusion:HR, *n* = 5–6 mice.

Considering evidence of a role for vascular Kvβ proteins in the adaptive response to exercise, we next tested the contribution of Kvβ to the regulation of myocardial perfusion after exercise training. To do so, we used myocardial contrast echocardiography (MCE; see Figure 3A) to quantify *in vivo* myocardial perfusion in sedentary and exercise-trained mice using a burst-replenishment ultrasound imaging protocol. Figure 3B shows exemplary plots of contrast signal intensity replenishment in sedentary and exercise-trained mice at rest and after moderate dobutamine-evoked stress. MCE revealed significantly greater myocardial perfusion in exercise-trained mice during stress (Figures 3B,C). Whereas differences in perfusion could be a consequence of differential cardiac workload and oxygen demand, we normalized perfusion to heart rate recorded during rest and stress periods. Accordingly, exercise-trained mice exhibited a significantly enhanced relationship between myocardial perfusion and heart rate relative to sedentary mice (Figure 3D). We observed similar responses to exercise training in male and female mice (Figure 3D and Supplementary Figure S1). Histological analyses revealed that myocardial capillary density in exercise-trained mice was not elevated (Supplementary Figure S2). These findings are consistent with previous work showing that chronic exercise promotes adaptive regulation of blood flow that supports enhanced oxygen delivery (31).

We next examined whether exercise impacts the molecular identity of the Kvβ complex in coronary arterial smooth muscle cells. To do this, we used *in situ* proximity ligation to quantify relative changes in Kv protein:protein interactions, as we have previously reported (19, 29). Consistent with the notion that exercise promotes remodeling of the vascular Kvβ complex, we observed a significant increase in Kv1.5:Kvβ2 interactions in coronary arterial smooth muscle isolated from exercise-trained mice relative to those from sedentary mice (Figures 3E,F). No differences were observed in Kv1.5:Kvβ1 interactions between exercise-trained and sedentary mice (Figures 3E,F), suggesting that exercise increases Kvβ2:β1 ratio in coronary arterial smooth muscle.

Based on the abovementioned findings, we repeated MCE measurements in exercise-trained and sedentary β2^−/−^ mice. Consistent with our previous study (17), sedentary β2^−/−^ mice showed suppressed myocardial perfusion during stress (Figure 3G). In stark contrast to observations in wt mice, the relationship between perfusion and heart rate was unchanged in exercise-trained β2^−/−^ mice (Figure 3G). Moreover, we measured resting perfusion in rtTA-Kcnab1 and control rtTA mice. As shown in Figure 3H, perfusion as a function of heart rate was enhanced in exercise-trained rtTA mice, but not in rtTA:Kcnab1 mice. These findings, taken together, are consistent with a central role for vascular Kvβ2 proteins in mediating enhancement of the relationship between perfusion and cardiac workload in response to chronic exercise.

### Kvβ proteins enable physiologic cardiac growth in exercise-trained mice

A comparison of echocardiography parameters in wt and β2^−/−^ sedentary and exercise-trained mice is provided in Table 2. Notably, systolic chamber diameters at systole were significantly lower in exercise-trained wt, but not β2^−/−^ mice relative to genotype-matched sedentary controls, suggesting mild systolic impairment in β2^−/−^ mice after exercise. Moreover, myocardial wall thickness (i.e., LVPWd/s, RWT) was significantly greater in wt exercise-trained mice relative to sedentary control mice, yet this effect was not observed in β2^−/−^ mice (Table 2). This was recapitulated in transgenic mice, whereas left ventricular mass measured via echocardiography was greater in exercise-trained rtTA mice (exercise: 132 ± 8 mg, sedentary: 109 ± 8 mg; *p* = 0.088; *n* = 4­­–5 mice) but not in rtTA-Kcnab1 mice (exercise: 109 ± 6 mg, sedentary: 119 ± 7 mg; *p* = 0.287; *n* = 7 mice each).

**Table 2:** Comparison of echocardiographic parameters in wild-type and Kvβ2^−/−^ exercised and sedentary mice.

Measurement	Wild-type		Kvβ2^−/−^	
	Sedentary	Exercise	Sedentary	Exercise
HR (BPM)	415 ± 7	424 ± 6	411 ± 10	438 ± 10
Endocardial values				
EDV (µl)	40.3 ± 2.6	37.8 ± 2.2	41.6 ± 1.6	40.0 ± 1.9
ESV (µl)	13.0 ± 1.2	10.7 ± 0.6	16.3 ± 1.4	12.5 ± 0.7 *
SV (µl)	27.3 ± 1.9	27.3 ± 1.9	25.5 ± 1.4	27.7 ± 1.8
EF (%)	67.5 ± 1.9	71.5 ± 1.1	61.3 ± 2.6	68.5 ± 1.9 *
FS (%)	36.2 ± 1.5	44.4 ± 1.9 **	35.3 ± 1.8	41.7 ± 1.7 *
CO (ml/min)	11.3 ± 0.6	11.5 ± 0.8	10.5 ± 0.7	12.1 ± 0.8
Chamber diameter				
LVIDd (mm)	3.7 ± 0.1	3.6 ± 0.1	3.5 ± 0.1	3.6 ± 0.1
LVIDs (mm)	2.4 ± 0.1	2.0 ± 0.1 *	2.3 ± 0.1	2.1 ± 0.1
Wall Thickness				
LVPWd (mm)	0.92 ± 0.06	1.25 ± 0.11 **	0.89 ± 0.04	0.83 ± 0.08
LVPWs (mm)	1.32 ± 0.03	1.65 ± 0.08 **	1.29 ± 0.04	1.32 ± 0.07
LVAWd (mm)	1.08 ± 0.06	1.13 ± 0.08	1.01 ± 0.05	1.10 ± 0.04
LVAWs (mm)	1.52 ± 0.07	1.65 ± 0.08	1.40 ± 0.05	1.65 ± 0.06 *
RWT	0.49 ± 0.04	0.70 ± 0.07 *	0.51 ± 0.03	0.47 ± 0.05

HR, heart rate; BPM, beats per minute; EDV, end diastolic volume; ESV, end systolic volume; SV, stroke volume; EF, ejection fraction; FS, fractional shortening; CO, cardiac output; LVIDd, left ventricular inner diameter at diastole; LVIDs, left ventricular inner diameter at systole; LVPWd, left ventricular posterior wall at diastole; LVPWs, left ventricular posterior wall at systole; LVAWd, left ventricular anterior wall at diastole; LVAWs, left ventricular anterior wall at systole; RWT, relative wall thickness.

* *p* < 0.05.

** *p* < 0.01, one-way ANOVA with Šídák's multiple comparisons test, *n* = 6–8 mice.

**Figure 4:**
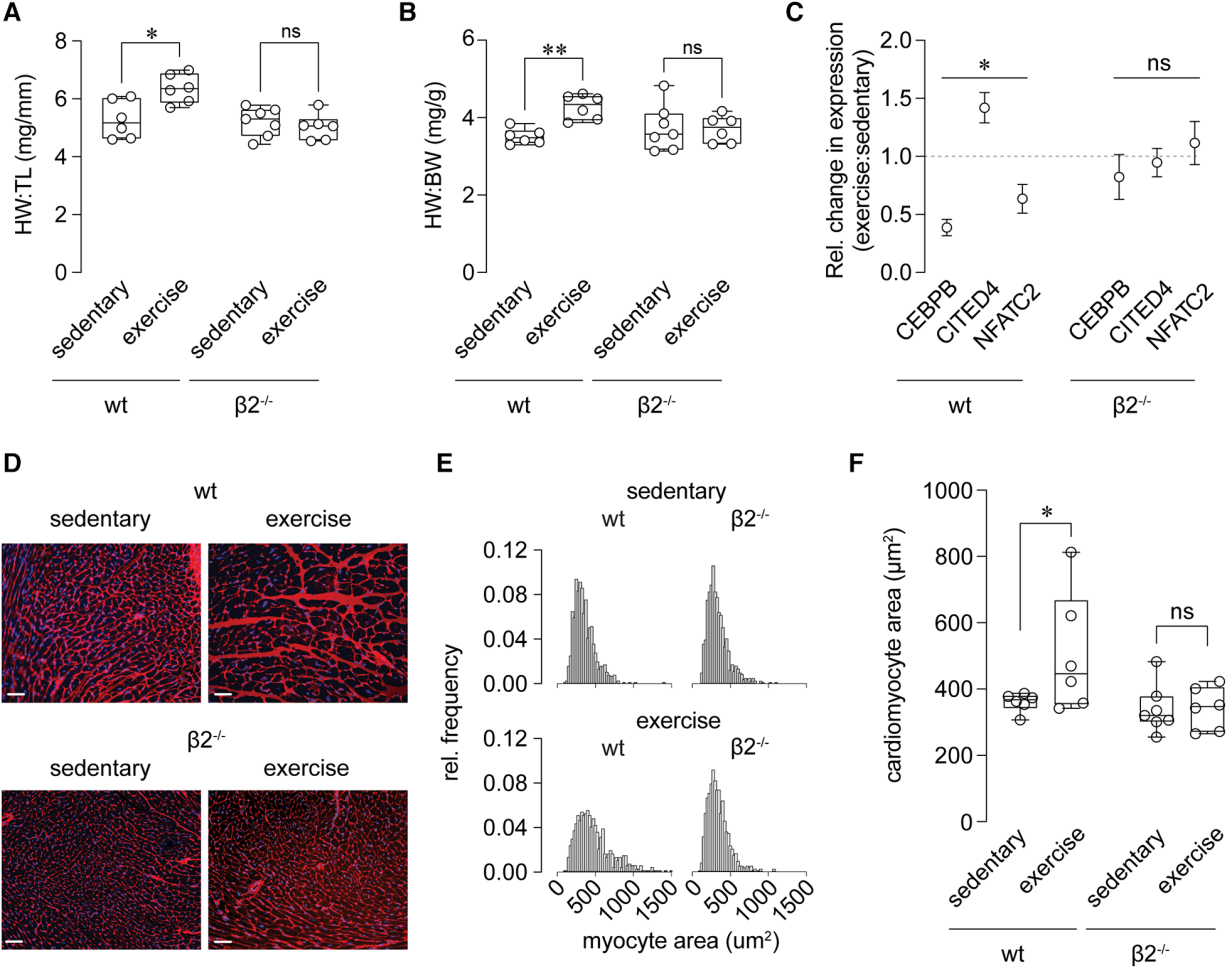
Abolished exercise-induced cardiac growth in β2^−/−^ mice. (**A**, **B**) Summary of heart weight (HW), normalized to tibia length (TL; **A**) or body weight (BW; **B**), determined by gravimetric measurements at necropsy of sedentary and exercise-trained wild-type (wt) and β2^−/−^ mice. *p < 0.05, ns: *p* > 0.05 (**A**); **p < 0.01, ns: *p* > 0.05 (**B**), wt exercise vs. sedentary, one-way ANOVA with Dunn's multiple comparisons test, *n* = 6–7 mice. (**C**) Summary of relative change in expression (exercise vs. sedentary) of CEBPB, CITED4, and NFATC2 transcripts in hearts from wt and β2^−/−^ mice. *p < 0.05, ns: *p* > 0.05, Wilcoxon test. (**D**) Representative fluorescence images of transverse left ventricular sections from wt and β2^−/−^ mice stained with alexa fluor 555-conjugated wheat germ agglutinin (membrane) and DAPI (nuclei). Scale bars represent 20 μm. (**E**) Relative frequency distributions showing myocyte cross-sectional areas in hearts from sedentary and exercised wt and β2^−/−^ mice. *n* = 665–811 total myocytes from 6 to 8 mice. (**F**) Summary of cardiac myocyte cross-sectional area in wt and β2^−/−^ sedentary and exercise-trained mice. *p > 0.05, ns: *p* > 0.05, one-way ANOVA with Holm-Šídák's multiple comparisons test *n* = 6–8 mice.

A fundamental adaptive cardiovascular response to exercise is cardiac hypertrophy, which reduces ventricular wall stress and promotes enhanced mechanical performance of the conditioned heart. We postulated that defective regulation of myocardial perfusion during stress upon modification of the Kvβ complex may perturb the growth response of the heart to exercise. Consistent with this, heart weight normalized to tibia length or body weight was significantly elevated in exercise-trained wt mice relative to sedentary control wt mice, yet this difference was absent in β2^−/−^ mice (Figures 4A,B). Furthermore, transcripts for biochemical markers of physiologic (i.e., CEBPB and CITED4) and pathologic (NFATc2) hypertrophy were significantly altered in hearts of exercise-trained mice (Figure 4C), consistent with previous work (32). These changes were abolished in exercise-trained β2^−/−^ mice.

To evaluate further differential cardiac growth responses, we performed a histological evaluation of cardiomyocyte size in exercise-trained vs. sedentary mice. As shown in Figures 4D–F and consistent with the aforementioned findings, exercise promoted an increase in cardiomyocyte cross-sectional area in wt mice, but failed to do so in β2^−/−^_._ These results suggest that, despite similar compliance to a 4 wk modest intensity training protocol between mice of both genotypes (see Figure 2B), mice lacking Kvβ2 proteins have an impaired cardiac hypertrophic response to long-term exercise training.

## Discussion

Here, we present evidence that cardiovascular Kvβ proteins influence exercise performance and facilitate adaptive responses to recurrent exercise training. The following novel findings support this conclusion: (1) mice lacking Kvβ2 proteins exhibited reduced exercise capacity, yet showed no discernible differences in lean or fat mass, diurnal activity patterns, or systemic metabolism; (2) despite compliance to a long-term exercise training regimen (4 weeks of running), β2^−/−^ mice failed to show improvements in exercise capacity; (3) exercise-trained wt mice showed enhanced myocardial perfusion under stress that was linked to an increase in Kv1.5:Kvβ2 protein interactions. Deletion of Kvβ2 abolished differences in myocardial perfusion following exercise training; (4) whereas wt mice subjected to a moderate-intensity running protocol demonstrated cardiac hypertrophy, this effect was absent in mice lacking Kvβ2; and, (5) the impacts of global deletion of Kvβ2 on exercise-induced enhancement in exercise capacity, augmentation of myocardial perfusion, and cardiac growth were recapitulated via selective increases in Kvβ1:β2 ratio in smooth muscle (i.e., rtTA-Kcnab1 mice). Overall, our results align with the notion that modulation of the Kvβ complex influences exercise performance and cardiovascular adaptations to long-term increases in physical activity, particularly through its regulation of vascular function and organ perfusion.

Our findings that Kvβ proteins influence physical performance and associated physiologic adaptations to long-term conditioning likely relate to well-established roles for voltage-gated potassium channels in controlling cardiovascular function. Multiple Kv channel subtypes underlie outward currents that regulate the repolarization phase of the cardiac action potential, and thereby influence the duration of the cardiac cycle. The dysregulation of cardiac Kv channels underlies electrical instability, arrhythmia, and cardiomyopathy (33). In the vasculature, members of several Kv channel families expressed in arterial smooth muscle cells control resistance vascular tone. The activity of vascular Kv channels opposes membrane potential depolarization and decreases Ca^2+^ influx via voltage-dependent Ca^2+^ channels, resulting in vasodilation and increased blood flow (34). Accordingly, functional impairment of Kv channels in the vasculature contributes to pathological conditions including hypertension and other ischemic disorders (35).

Native Kv channels in the cardiovascular system form as transmembrane tetrameric structures that comprise a voltage-gated pore (18, 36). The pore domain interacts with a heteromeric intracellular Kvβ complex (37), which regulates subcellular distribution and gating kinetics, adding further diversity to native Kv currents among excitable cells (38). One intriguing aspect of Kvβ function relates to a potential role in redox sensing that links intermediary metabolic processes with membrane excitability (38, 39). Prior work has shown that as members of the aldo-keto reductase superfamily, the Kvβ proteins bind nicotinamide adenine dinucleotide cofactors [i.e., NAD(P)H] (40). Our recent work demonstrated that Kvβ redox sensitivity underlies the physiological control of organ perfusion (22). In response to catecholamine-induced stress, heightened myocardial oxygen demand, via a yet unknown mechanism, evokes a change in redox potential in coronary arterial smooth muscle that culminates in increased cytosolic NADH:NAD ^+ ^. This change in pyridine nucleotide redox state in smooth muscle is sufficient to augment outward K^+^ current via Kv1 channels in a manner that requires both the presence and intact catalytic function of Kvβ2. Hence, Kvβ catalytic function appears to be coupled to channel gating and membrane potential regulation in the vasculature. Nonetheless, how changes in enzymatic activity of the Kvβ proteins upon cytosolic elevation of carbonyl substrate concentrations and/or redox potential influences Kv gating warrants further investigation.

Whereas the Kvβ complex in cardiac and vascular smooth muscle consists of both Kvβ1 and Kvβ2 proteins, here we show that the deletion of Kvβ2 suppresses exercise capacity. Prior echocardiographic analyses of β2^−/−^ mice demonstrated a time-dependent decline in mean arterial pressure during norepinephrine-induced stress, suggesting a role in supporting cardiac contractile performance during conditions of elevated catecholamines (17). Our current work suggests that suppressed myocardial perfusion in β2^−/−^ animals, both at baseline and after training, may hamper organ perfusion and exercise capacity. This finding is in line with human studies in patients with diminished flow reserve due to microvascular disease and inadequate myocardial oxygen delivery. Patients with microvascular disease and low coronary flow reserve have greater inducible ischemia during exercise compared with controls with normal flow reserve (41). Moreover, patients with angina and coronary microvascular disease have markedly reduced VO_2_ max and exercise capacity compared with sex-matched control subjects (42). Interestingly, these patients also had markedly reduced peak heart rate responses to exercise and heart rate recovery, suggesting that a mismatch between oxygen supply and demand may diminish chronotropic responses during exercise and thus limit physical performance. Our results suggest that impaired redox-dependent coronary vasodilation in response to increased workload could at least partially underlie the link between inadequate oxygen delivery and exercise intolerance, and further implicate vascular Kvβ proteins as potential therapeutic targets.

Upon subjecting mice to a moderate-intensity long-term exercise regimen, mice lacking Kvβ2 were able to complete a similar quantity of training activity compared with wt controls. Nonetheless, the β2^−/−^ mice diverged with wt controls with respect to adaptations to long-term training. This includes failure to improve in the level of work performed in exercise capacity tests relative to baseline capacity, a lack of enhancement of myocardial perfusion during dobutamine-evoked cardiac stress, and an abolished cardiac hypertrophic response. It is plausible that the phenotypic links between myocardial perfusion, cardiac growth, and exercise performance observed in this study stem from altered vasoregulation and limited oxygen delivery to the heart and peripheral tissues upon loss of Kvβ proteins. During exercise, the heart consumes large quantities of ATP to support contractile protein function and maintain ionic homeostasis (43). Mice lacking Kvβ2 proteins or overexpressing Kvβ1 proteins in smooth muscle (i.e., elevated Kvβ1:β2 ratio) not only have attenuated hyperemic responses during stress (17), but also lack augmentation of the hyperemic response after training (see Figure 3). Our data are consistent with the concept that adaptations in myocardial perfusion, via upregulation of Kv1:Kvβ2 interactions, are essential for modifying oxygen delivery to the exercise-adapted heart. Moreover, in the absence of optimal oxygen delivery during stress, the physiologic growth program is blunted. While the question of whether these phenomena are causally linked remains unknown, recent work has highlighted the importance of cardiac metabolism to exercise-induced cardiac growth (44). It is thus plausible that mismatched oxygen supply and demand in animals with elevated vascular Kvβ1:β2 ratio promotes increased cardiomyocyte reliance on glucose as an energy source. This effect may limit biosynthetic pathways and the associated activation of transcriptional programs (e.g., CEBP/β, CITED4) that are known to drive physiologic cardiac hypertrophy (45).

Several limitations of the current study should be taken into consideration to contextualize our findings. Firstly, this study was conducted in mice, a model system that does not fully reflect human physiology, metabolism, or responses to prolonged exercise. While conducting this study in rodents provides valuable insights, the translation of results to human populations may be limited by these inherent disparities. Nonetheless, the use of mice allows for testing the effects of time- and intensity-controlled exercise in the absence of confounding environmental complexities such as dietary variations, psychosocial stressors, and xenobiotic exposures. Moreover, we acknowledge that Kvβ2 may have physiological roles beyond that of vascular regulation (e.g., autonomic balance, immunity) that may also impact exercise-induced tissue adaptations (46). Indeed, our conclusions are partially based on data acquired from mice with global deletion of Kvβ2, which is highly expressed in excitable cells outside of the cardiovascular system. For instance, considering prior work showing that Kvβ2-null mice exhibit altered skeletal muscle growth and function (47), we cannot rule out the potential contribution of skeletal muscle Kvβ2 to differences in exercise capacity at baseline and after training. However, despite these considerations, parallel observations between global Kvβ2-null mice and transgenic mice with inducible smooth muscle selective elevation of Kvβ2:β1 ratio reinforces the notion that the regulation of tissue perfusion by the Kvβ complex contributes, at least in part, to exercise-induced adaptations.

Our results shed light on Kvβ proteins as promising targets for enhancing exercise performance and potentially amplifying the beneficial effects of exercise on cardiovascular health. We speculate that the enzymatic properties of Kvβ and associated regulation of Kv function in the vasculature may underlie its influence on exercise outcomes. If this is the case, devising strategies to modulate Kvβ AKR function to augment redox sensitivity could facilitate a more efficient coupling between metabolism and vascular tone. This strategy could effectively improve oxygen delivery to the heart and other tissues (e.g., working skeletal muscles), consequently enhancing function during exercise. Introducing small molecules targeting Kv channels, particularly those aimed at modulating the AKR function of Kvβ2, thus holds promise for fine-tuning exercise responses and optimizing cardiovascular adaptations. Further work is warranted to elucidate the precise mechanisms involved and to explore the therapeutic potential of such interventions in improving exercise performance and cardiovascular health.

## Data Availability

The original contributions presented in the study are included in the article/**Supplementary Material**, further inquiries can be directed to the corresponding author.
