# Supplementary material for: Kv beta complex facilitates exercise-induced augmentation of myocardial perfusion and cardiac growth
Source: Front Cardiovasc Med. 2024 Jun 24;11:1411354. doi: 10.3389/fcvm.2024.1411354 (PMC11228310; doi:10.3389/fcvm.2024.1411354)
Supplement: Supplementary file 1 [file Datasheet1.docx]

**
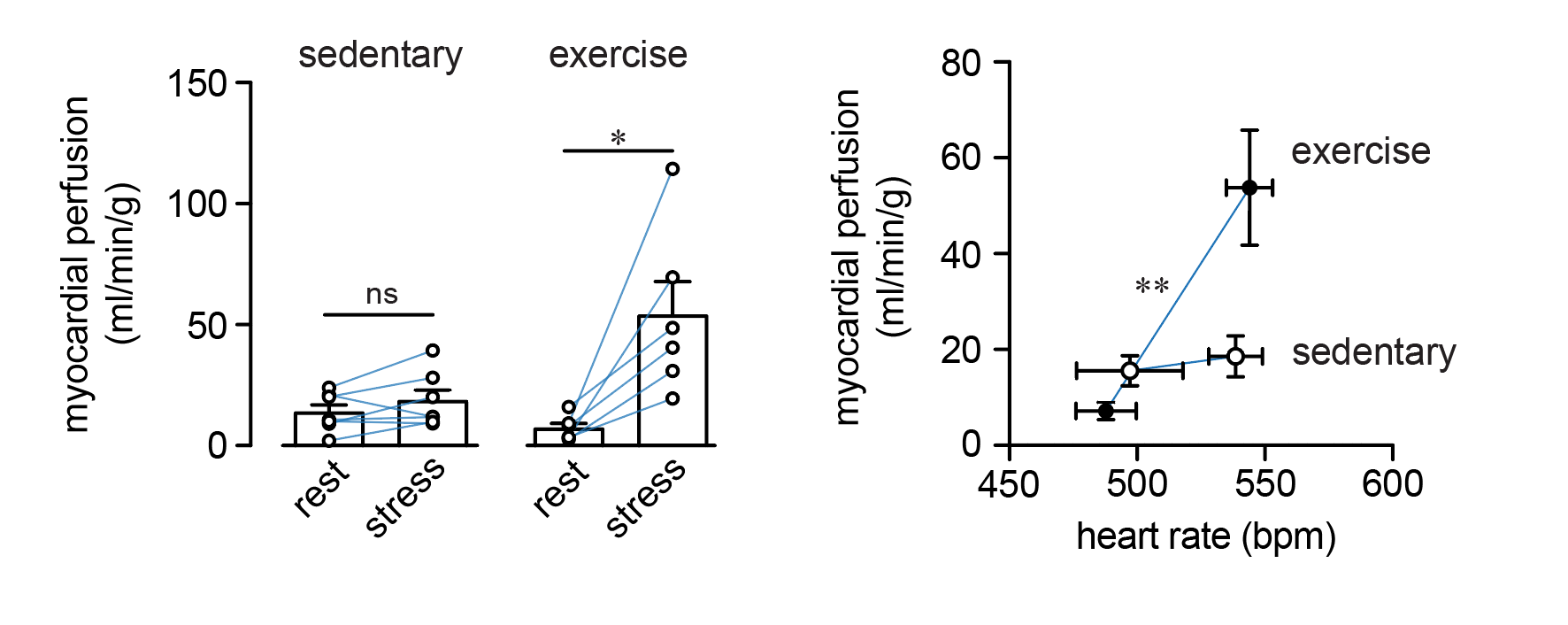
**

**Figure S1: Exercise enhances myocardial perfusion in female mice.** *Left:* Summary plot of myocardial perfusion at rest and during dobutamine-induced stress in sedentary and exercise-trained female mice. *p<0.05, ns: p>0.05, paired t-test. N=5–6 mice. *Right:* Relationships between myocardial perfusion and HR in sedentary and exercise-trained female mice at rest and stress. **p<0.01, mixed effects analysis on perfusion:HR, n=6–8 mice.


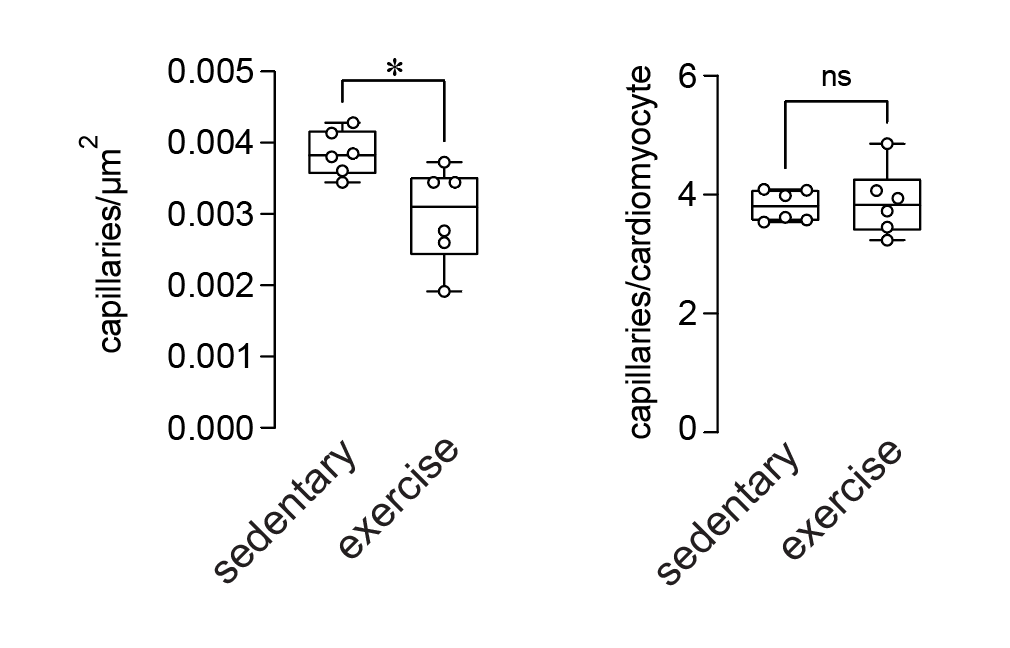


**Figure S2: Myocardial capillary density in sedentary and exercise-trained mice.** Summary of capillaries (positive isolectin B4 stain) per μm^2^ of ventricular tissue section (left) and capillaries per cardiomyocyte in hearts of sedentary versus exercise-trained wild-type male mice. *p<0.05, ns: p>0.05, Mann Whitney test, n=6 mice each.
